# Pulse Duration and Wavelength Effects of Laser Ablation on the Oxidation, Hydrolysis, and Aging of Aluminum Nanoparticles in Water

**DOI:** 10.3390/nano9050767

**Published:** 2019-05-18

**Authors:** Ke Zhang, Dmitry S. Ivanov, Rashid A. Ganeev, Ganjaboy S. Boltaev, Pandiyalackal S. Krishnendu, Subhash C. Singh, Martin E. Garcia, Irina N. Zavestovskaya, Chunlei Guo

**Affiliations:** 1 The Guo China-US Photonics Laboratory, State Key Laboratory of Applied Optics, Changchun Institute of Optics, Fine Mechanics, and Physics, Chinese Academy of Sciences, Changchun 130033, China; zhangke16@mails.ucas.ac.cn (K.Z.); rashid_ganeev@mail.ru (R.A.G.); ganjaboy_boltaev@mail.ru (G.S.B.); india6krishna@gmail.com (P.S.K.); ssingh49@UR.Rochester.edu (S.C.S.); 2 University of Chinese Academy of Sciences, Beijing 100049, China; 3 Theoretical Physics Department, University of Kassel, 34132 Kassel, Germany; ivanov@uni-kasseel.de (D.S.I.); m.garcia@uni-kassel.de (M.E.G.); 4 National Research Nuclear University MEPhI (Moscow Engineering Physics Institute), 115409 Moscow, Russia; INZavestovskaya@mephi.ru; 5 The Institute of Optics, University of Rochester, Rochester, NY 14627, USA

**Keywords:** pulsed laser ablation, aluminum, alumina, aluminum hydroxide, aging, morphology, molecular dynamics, two temperature model, modeling and simulations

## Abstract

We analyzed the formation of the aluminum (Al) nanoparticles (NPs) with triangular shape obtained by ablating Al bulk in liquid using pulses with different durations (5 ns, 200 ps, and 30 fs) and wavelengths (355 nm, 800 nm, and 1064 nm). We report three stages of synthesis and aging of Al NPs: Formation, transformation, and stable stage. The NPs prepared by different pulses are almost identical at the initial stage. The effects of duration and wavelength of the ablation pulses on the aging of NPs are revealed. Pulse duration is determined to be essential for morphological transformation of NPs, while pulse wavelength strongly influences particle sizes. NPs produced by ultra-short pulses have smaller sizes and narrow size distribution. We demonstrate that oxidation and hydrolysis of Al in water are the results of ablation for all pulse durations and wavelengths, which also strongly modify the preferable reaction path of NPs in water, thus affecting the composition and morphology of triangle NPs. The results of modeling of the NPs generation in water due to a 50 ps laser pulse interacting with a thick Al target are presented. Water-based effects in the formation of NPs, their evolution, and solidification are considered from the mechanical and thermophysical points of view. The detailed analysis of the modeling results allowed for determination of the main mechanism responsible for the ablation process followed by the NPs formation.

## 1. Introduction

Nanoparticles (NPs) attract significant interest because of special properties and various applications [1,2,3,4,5]. For example, Al NPs have a role in propellants and pyrotechnics, because they can provide additional heat release during exothermic oxidation [6,7]. The optical character of Al NPs also contributes to their application for solar cells [8], optoelectronic devices [9], metal-enhanced fluorescence devices [10], and broad-band wire-grid polarizers [11]. In addition, the search for renewable energy sources and the development of the hydrogen production industry have prompted researchers to focus on spontaneous thermal reaction based on aluminum–water systems [12,13]. Al nanostructures can also be used as components of high-capacity hydrogen storage materials due to the fact that hydrogen can be generated through the reaction between aluminum and water [14,15,16].

Aluminum, a very active metal, can spontaneously react with oxygen or water from room temperature up to its melting temperature (660 °C) Normally, a dense oxide film (aluminum oxide, alumina, Al_2_O_3_) is formed by the reaction of Al with oxygen, which prevents other reactions. Chemical reaction between Al and water is so slow that it is virtually unobservable. Aluminum hydroxide [Al(OH)_3_], which will be formed at the initial stage, can entirely isolate the contact between Al and water. However, the chemical reactions are very different once one considers the nano- and macro-scale structures [17,18,19]. In the case of nanoscale, studies have shown that morphology [20,21] and sizes of particles [22,23,24] have an impact on their chemical properties. Therefore, it is worth studying the dynamics of morphology and particle sizes of Al, aluminum oxide, and hydrolysates at the nanometer level.

The performance of materials at nanoscale is totally different from their macroscopic performance. The mixture of Al powder and Al oxide powder can significantly improve the efficiency of hydrogen production [13]. Due to the enormous industrial importance of hydrogen, there has been extensive research on Al–water systems, mainly including interactions with pure water systems [25], acid-based systems [13], oxide systems [26,27], and salt systems [13]. What these studies have in common is the destruction of the oxide layer to facilitate the continuation of the reaction. Particularly, the syntheses of Al and its oxide NPs by pulsed laser ablation in liquid (PLAL) was considered to be an efficient, simple, and green method [28]. In addition, this method has been widely used in the syntheses of other metal and oxide NPs [29,30,31,32,33]. However, many factors, such as the type of surrounding media [34,35,36], temperature of liquid [37,38,39], laser pulse duration [40] and fluence [41,42], the relative position of the focus and the target surface [43], adding surfactant [44], and characteristics of targets [45,46], strongly influence the ablation processes in such a manner that the composition, morphology, and particle sizes of synthesized NPs show significant differences under different conditions.

In order to support the results of our experimental studies and to take a deeper view on the process of formation of Al nanoparticles in liquid media at the atomic level, the molecular dynamics (MD)-based simulations and two temperature model (TTM) were used [47]. The essential concepts of such combined MD-TTM are described in Reference [48]. The hybrid atomistic-continuum model MD-TTM addresses the kinetics of fast laser-induced non-equilibrium phase transitions at the atomic level using MD approach. Based on the developed approach, the MD-TTM model is further modified for multiprocessing mode with a message passing interface (MPI) algorithm for applications in simulations on nanostructuring of metals and generation of NPs in air and in water on the experimental scale. The model is described in detail in Reference [49] for the case of nanostructuring of thick metal targets with an UV-laser pulse, whereas its successful applications for the case of thin targets were reported in References [50,51].

In this paper, the ablation of Al targets in distilled water by 800 nm laser pulses of different durations (5 ns, 200 ps, and 30 fs) is analyzed along with the study of the influence of different wavelengths (1064 nm and 355 nm) of the heating radiation on this process. It is found that the morphology of NPs prepared under different pulse durations changes significantly over time. We demonstrate how the pulse duration influences the aging of NPs. In addition, the compositions of NPs obtained using different durations and wavelengths of heating pulses are found to vary significantly. We also present the results of modeling of the NPs formation in water media using 50 ps laser pulses interacting with a thick Al target. This duration is much longer than the characteristic electron-phonon relaxation time for Al (τ_e-ph_~1.5 ps) [52], which makes our modeling results identical to that if we used 200 ps. At the same time, the shorter pulse leaves the efficiency of our computations high enough. The performed modeling enables a detailed analysis of the mechanism of NPs formation in water due to the laser heating of Al, which allows for drawing a conclusion about the effect of liquid environment on the formation of NPs, their evolution, and solidification process.

## 2. Experimental

The experiment was accomplished by irradiating the Al target in distilled water with focused laser pulses. A sheet of bulk Al (99.999%, ZhongNuo Advanced Material Technology Co., Limited, Beijing, China) was polished and cleaned in anhydrous alcohol and then placed in a quartz cell (30 × 30 × 30 mm^3^) filled with distilled water, which can be moved along the two directions. Laser pulses were focused by a 150 mm focal length lens on the surface of bulk Al. The beam diameter on the ablating surface was 0.3 mm. Details of the experimental setup are given elsewhere [53,54,55].

The ablation was carried out using the Ti: Sapphire laser (Spitfire Ace, Spectra Physics, Santa Clara, CA, USA), which provided the 800 nm, 1 kHz pulses of 30 fs and 200 ps duration. The nanosecond laser (λ = 1064 nm, τ = 5 ns, 10 Hz pulse repetition rate; Q-Smart, Coherent, Santa Clara, CA, USA) and its third harmonic (λ = 355 nm) were also used for ablation. Laser ablation was carried out using 48 mJ, 0.56 mJ, and 0.68 mJ energies of ns (nanosecond, 1064 and 355 nm), picosecond (ps), and femtosecond (fs) pulses, respectively. Each ablation process lasted for 30 min. The synthesized NPs suspensions are designated as S_n(1064/355)_, S_p_, and S_f_ for 5 ns, 200 ps, and 30 fs ablation processes, respectively.

Sizes, macrostructure, and morphology of NPs were analyzed by scanning electron microscope (SEM; S-4800, Hitachi, Tokyo, Japan), which allowed determination of the change of synthesized particles morphology during a long period. Composition and structure of NPs were determined by energy dispersive spectroscopy (EDS; S-4800, Hitachi) and X-ray diffraction (XRD; D8 Discover, Bruker AXS, Karlsruhe, Germany). UV-vis-NIR absorption spectroscopy (Cary Series, Agilent Technologies, Santa Clara, CA, USA) was used to determine the optical bandgaps of suspensions and to monitor the number of NPs in the water colloids.

## 3. Results and Discussion

### 3.1. Color and Spectral Variations of ns-, ps-, and fs-Induced Al Suspensions

The ablation of Al in water under various conditions showed that pulse duration and wavelength have a close connection on morphology, size, and composition of NPs, which were changed during some period after ablation. These studies showed that pulse duration significantly influences the modification of synthesized particles.

Thin layers of Al_2_O_3_ and Al(OH)_3_ produced during initial stages of interaction of Al, oxygen, and water suppressed further interaction between those components. Laser irradiation destroyed those films and allowed the reactions to continue. In addition, the heating effect of laser–matter interaction also played a role in promoting the reaction. The equations for Al to react with oxygen and water at room temperature are as follows:

(1) $$4\mathrm{Al}+3O_{2}=2{\mathrm{Al}}_{2}O_{3}$$

(2) $$2\mathrm{Al}+6H_{2}O=2\mathrm{Al}{\left(\mathrm{OH}\right)}_{3}+3H_{2}$$

The favorable temperature range for producing Al(OH)_3_ lies between room temperature to 280 °C. Above 480 °C, Al_2_O_3_ becomes the most probable product of reaction according to previous studies [26,27], which, however, did not take into account the influence of pulsed laser interaction. Extreme conditions of reaction, such as high temperature and high pressure provided by laser radiation, play a decisive role during the process of ablation [56]. It was shown previously that the external environment affects the self-organization process of bubbles in the corrosion process [57]. The plume induced by laser rapidly cools down in the solution that led to the formation of NPs through agglomeration. Agglomeration not only influences the whole process of NPs formation, but also plays an important role in the subsequent aging processes.

Earlier, Al and Al_2_O_3_ complexes were synthesized using ablation by pulsed Nd:YAG laser in liquid environment without any surfactant or catalyst in deionized water [58]. It was found in another study that Al(OH)_3_ produces when the surfactant, such as cetyl trimethyl ammonium bromide, becomes added to the water [59]. Meanwhile, it was found during our laser ablation studies that, without any surfactant or catalyst, Al_2_O_3_ and Al(OH)_3_ coexist during the process of ablation, while pulse duration significantly influences their relative content.

After polishing, the pure Al was placed in the distilled water. It reacts rapidly with oxygen and water, forming a film containing Al_2_O_3_ and Al(OH)_3_. The laser pulses destroy this film, re-expose pure Al, and allow further reactions between above components. The pulse heating also promotes the reaction between them. Therefore, the NPs containing Al, Al_2_O_3_, and Al(OH)_3_ should appear in the solution during ablation at the same time. This assumption has been proven during structural and morphology analysis of synthesized species.

To analyze the role of pulse duration during formation of NPs, laser pulses with different durations were used to ablate the target. The influence of fundamental and third harmonic wavelengths on ablation was compared under ns ablation conditions. The suspensions obtained during ablation of Al bulk with variable pulse durations were slightly different in color. The colloidal solution obtained under ablation by ns pulses showed milky white color, while ps radiation caused the light gray color and the colloidal solution produced by fs pulses ablation showed a brown color. In all of the above-mentioned cases, the solutions had the micro-emulsion consistence, and after a few days, the precipitation started to play important role.

**Figure 1:**
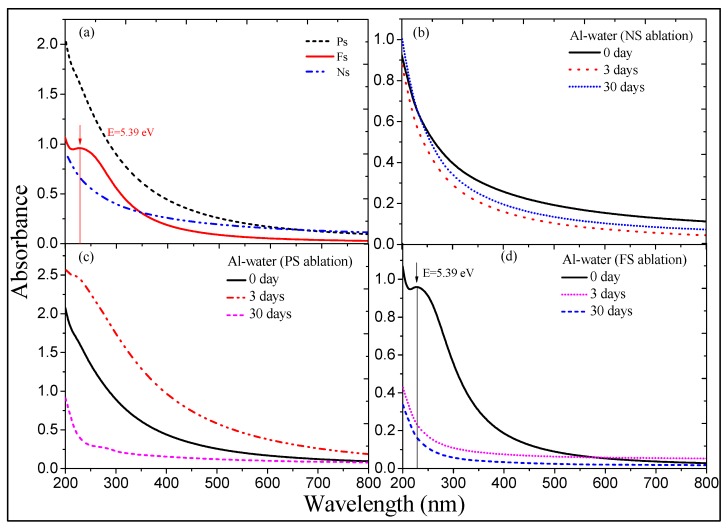
Absorption spectra of Al nanoparticles (NPs) colloidal solutions. (**a**) Absorption spectra measured immediately after ablation using different pulse durations (Ns: 5 ns, 1064 nm, 10 Hz, 48 mJ; Ps: 200 ps, 800 nm, 1 kHz, 0.56 mJ; Fs: 30 fs, 800 nm, 1 kHz, 0.68 mJ). (**b**) Dynamics of absorption spectra of aged NPs in the case of ablation by ns pulses. (**c**) Absorption spectra variations in the case of ablation by ps pulses. (**d**) Variation of absorption of the NPs suspension prepared using fs pulses.

Absorption spectra of the colloidal solutions obtained by PLAL were measured immediately after ablation. The results are shown in Figure 1a. The solutions exhibited large absorption in the ultraviolet range, and the edge of absorption band extended towards the visible region, so that the solutions had different colors. The suspension of NPs prepared by fs pulses showed a resonance peak located at λ = 230 nm (E = 5.39 eV, red curve), while there were no similar peaks in the case of application of the ns (dot-dashed curve) and ps (dashed curve) pulses. Earlier, 150 ps pulses of Nd:YAG laser were used to ablate Al target in ethanol, and the colloidal solution had a similar absorption peak, which was explained as the product of ethanol decomposition [60]. Compared with their research, different pulse duration (30 fs) and solvent (distilled water) were used in our study, which showed the same peak of absorption. Therefore, the appearance of the peak can be considered independent of the pulse duration and solvent used, but rather attributed to the peculiarities of the products of ablation of Al target.

In addition, during ablation by fs pulses, a bright white light was emitted when ultra-short pulses passed through the liquid. Extremely high peak power led to stronger absorption of the laser radiation passing through the liquid, thus reducing the intensity at the target surface, which has earlier showed the low rate of NPs formation by fs pulses [60]. As a result, the absorption of these colloids was significantly reduced compared with the former case. The repetition rate of ns pulses (10 Hz) was significantly smaller, and the pulses acting on the bulk surface during the same time were 100 times less compared with the ablation by fs or ps pulses. Therefore, the number of NPs generated by excitation of ns pulses was significantly lower, which led to significantly smaller absorption (dot-dashed curve).

The colloidal solutions were also analyzed 3 and 30 days after the ablation (Figure 1b–d). The major changes in the morphology occurred mainly during the first few days after the ablation. Since then, the morphology and particle sizes remained basically stable, so no measurements of the absorption spectra of colloidal solutions during the stable period were carried out. The colloidal solution S_n_ did not show essential changes in the absorption spectrum (Figure 1b). The stability of absorption spectrum in that case corroborated the SEM studies of Al NPs (see the following subsection). Meanwhile, the absorption spectra of colloidal solutions S_p_ (Figure 1c) and S_f_ (Figure 1d) have undergone significant changes. The absorption of S_p_ was slightly increased on the third day and a “shoulder” appeared at λ = 240 nm (E = 5.17 eV) compared with the initial colloidal solution. This modification was mainly based on the difference of NPs’ morphology. The absorption spectrum measured after 30 days was further modified, and the position of shoulder became red shifted. The remarkable difference between the 3rd and the 30th days indicates that the morphological structure of particles is still changing at this stage. Other test results also showed that the colloidal solution obtained using ps pulses-induced ablation is the most surprising and complex. Spectroscopic measurements of the solution S_f_ showed that, after the third day, its properties became almost stabilized.

### 3.2. Morphology of Synthesized Al NPs

SEMs of three groups of colloids were performed to determine the morphology, particle sizes, and distribution of NPs. For ns pulses, ablation was operated at two wavelengths (1064 nm and 355 nm). These studies allowed analyzing the influence of the wavelength of 5 ns ablation beam on the formation of NPs. SEM of ablation showed that the initial morphology of NPs was close to the regular spherical structure. The NP structures obtained under different wavelength conditions (1064 nm and 355 nm) did not distinguish from each other.

**Figure 2:**
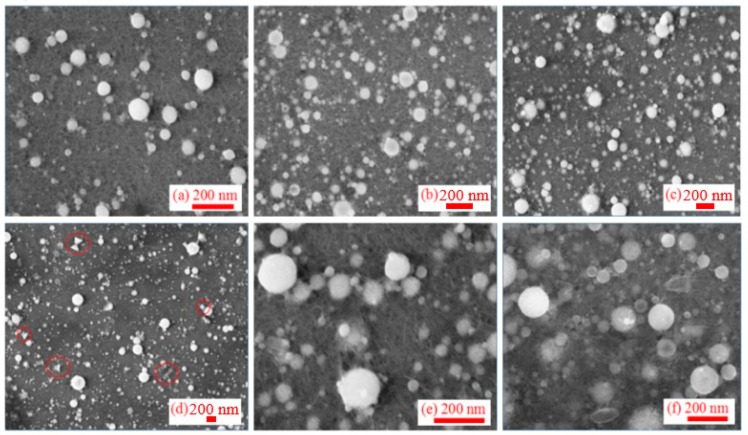
SEMs of the colloidal solutions synthesized using ns, 1064 nm pulses and measured at different time intervals from ablation: (**a**) Less than 1 day after ablation, (**b**) 8 days, (**c**) 15 days, (**d**) 40 days, (**e**) 60 days, and (**f**) 90 days after ablation.

In the case of 1064 nm pulses, SEM images did not show significant difference in morphology and particle sizes during the aging process for more than three months (Figure 2). Compared with other metal NPs [61], the Al NPs obtained at these conditions of ablation did not show the aggregation. On the 40th day (Figure 2d), when the same colloidal solution of NPs was measured, it was found that the morphology of very few NPs presented the conical or rod-like structures (marked by red rings), while most of NPs showed the regular spherical shape.

**Figure 3:**
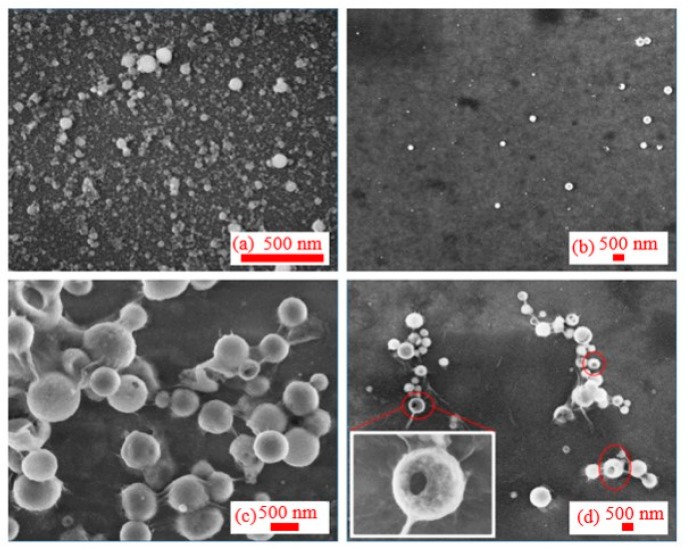
SEM images at different periods after ablation by 355 nm radiation: (**a**) 1 day, (**b**) 20 days, (**c**) 45 days, and (**d**) 60 days after ablation, inner image: individual particles show "red blood cells" structure due to aging.

In the case of ablation using 355 nm, 5 ns pulses, the initial shapes of NPs were similar to the case of ablation using 1064 nm radiation (Figure 3). The size of particles significantly increased after 60 days from ablation. SEM studies of the newly synthesized NP solution showed the presence of a powdery structure uniformly distributed on the substrate of silicon wafer. After 30 days, the surrounding powders aggregated and coagulated to form new particles with notably larger sizes. Smaller particles and colloids in liquids adhered to the surface of NPs and then coagulated with them (Figure 3c). The NPs still have spherical structure. Additionally, two months later, the test results showed that there were a few holes on the surfaces of some NPs, which had a doughnut-like structure (Figure 3d).

**Figure 4:**
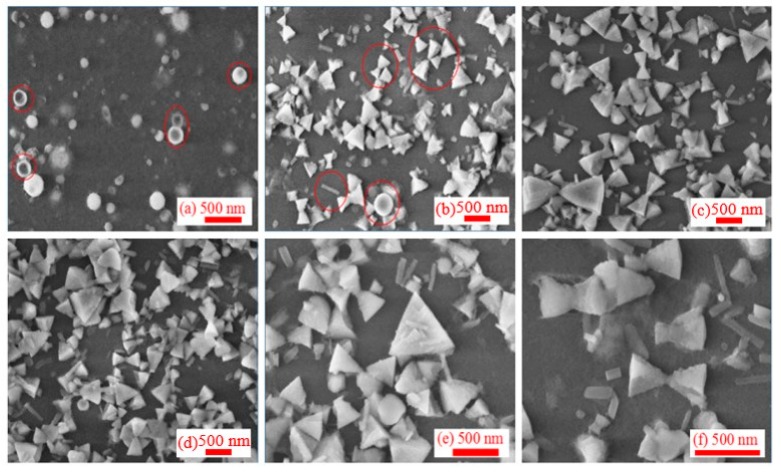
SEM images of NPs at different stages after ablation by 200 ps, 800 nm, 0.54 mJ pulses, (**a**) 1 day, (**b**) 10 days, (**c**) 30 days, (**d**) 60 days, (**e**) 90 days, and (**f**) 100 days after ablation.

The ablation of Al by ps pulses is a commonly used technique. However, the most interesting stage of the morphology dynamics at the conditions of ablation has not yet been analyzed, to the best of our knowledge. SEM images of NPs at different stages after ablation by ps pulses are presented in Figure 4. SEM of initial NPs showed the spherical shapes and narrow particle size distribution. Some of them showed the structure similar to the red blood cells (Figure 4a). Meanwhile, the morphology of NPs was notably changed during the following days. There were no transitional structures between initial (spherical) and subsequent (cone-shaped and triangle) morphologies. Therefore, we do not think that structures like “red blood cells” can be considered as the precursors of the subsequent morphology variations.

Figure 4b shows a typical SEM image obtained 10 days after ablation, when the spherical particles were transformed completely into a large number of triangles and conical structures supplemented by a small number of rod-like NPs and very rare spherical structures (see marked red rings). In the subsequent SEM measurements, the conical and rod structures remained stable, while the spherical structures completely disappeared (Figure 4c–f). Interestingly, many conical structures became joined in pairs to form bow-tie structures (Figure 4b). This unique structure makes the number of free electrons located at the apex of the cone significantly larger than in other positions.

**Figure 5:**
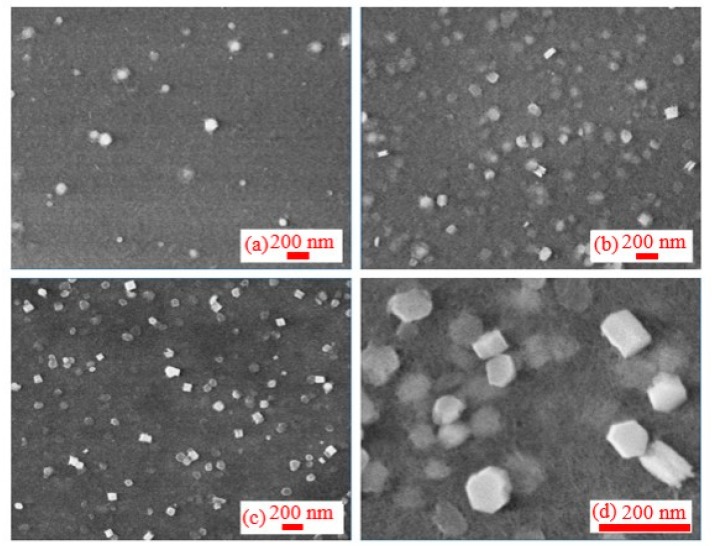
SEM images of NPs at different stages after ablation by 30 fs, 800 nm pulses: (**a**) within 1st day, (**b**) after 20 days, (**c**) after 40 days, and (**d**) after 80 days from ablation.

Results of ablation by fs pulses completely differ from the ps ablation. Although the initial NPs (Figure 5a) were similar to those in the previous two cases (ns and ps pluses) and showed regular globules with a narrow range of particle size distribution, the influence of pulse duration became fully recognizable during the subsequent aging process (Figure 5b–d). After a period of aging, its morphology presented the granular structures with uniform size distribution. Individual particles demonstrated the layered structure. Such a structure remained stable during the following three months of aging research.

We divided the life cycle of particles into three stages. (1) Formation stage (during ablation and within 24 h after ablation): Within a short time after ablation and during the process of ablation, the plume cooled down rapidly and formed the spherical NPs by aggregation. At this stage, the particles interact with the surrounding liquid environment, and the sizes, morphology, and composition of particles are still unstable. (2) Great change stage (a few days after ablation): This stage is a transitional period, when NPs continue interacting with surrounding environment and with each other, which causes the largest changes in their morphology. The sizes, morphology, and composition of NPs drastically vary during this stage, thereby gradually becoming more stable. (3) Stable stage (about three months after the completion of great change stage): This stage presents the final results of the ablation by pulsed lasers in the liquid environment. During this stage, the influence of external conditions on the ablation process, such as pulse duration and wavelength, diminishes. The duration of each stage of the same ablation process varies greatly. For Al target, the influence of pulse duration and wavelength on the morphology and sizes of synthesized particles is very significant, especially in the subsequent aging processes. The sizes of NPs obtained by ultra-short pulses were remarkably smaller and more homogeneous.

**Figure 6:**
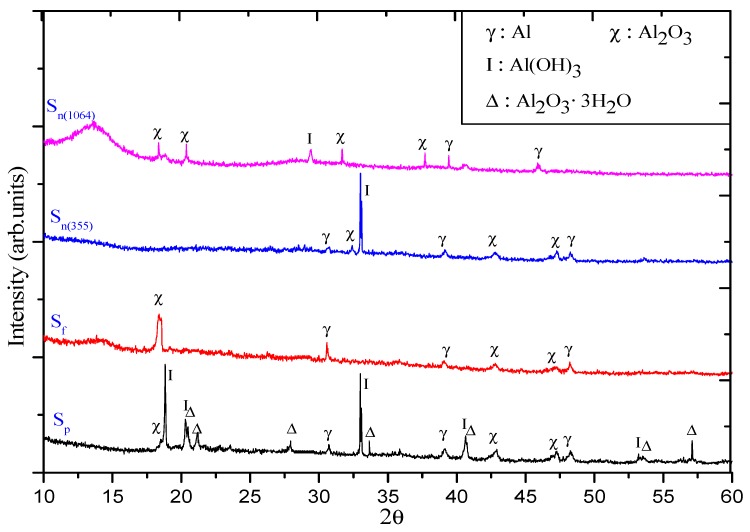
XRD image of NPs prepared at different conditions. S_n(1064)_: NPs obtained by 1064 nm, 5 ns pulses. S_n(355)_: NPs obtained by ns pulses with 355 nm. S_f_: NPs obtained by fs pulses. S_p_: NPs obtained by ps pulses.

EDS and XRD were carried out to further analyze the composition and structure of NPs obtained using different pulse durations and wavelengths. Considering the intense interaction between NPs and the surrounding environment during the formation and great change stages, the composition and structure of NPs are still unstable. Therefore, EDS and XRD were measured during the stable stage. Colloidal solutions were deposited and evaporated repeatedly in air at room temperature onto the silicon wafer with oxide film on its surface for EDS and XRD. XRD of samples are shown in Figure 6. The EDS test results of each sample are shown in Table 1.

It was found during EDS and XRD studies of the ns-induced suspension that this composition includes Al, Al_2_O_3_, and Al(OH)_3_. The peak intensity of each component is similar in the XRD images. In the case of 355 nm ablating pulses, Al(OH)_3_ showed a very strong peak compared to those from Al and Al_2_O_3_. The EDS and XRD of the suspensions produced by 1064 nm and 355 nm radiation were completely different. In the case of ps pulses, the NPs mainly show two different morphologies, the conical shape dominated by Al_2_O_3_ 3H_2_O and the rod shape dominated by Al(OH)_3_. In addition, Al still exists in NPs according to XRD. A specific morphology, such as conical shape, comprises Al_2_O_3_ 3H_2_O as the main component, while the NPs do not exist in the state of a single crystal. Compared with the former case, XRD peaks of NPs obtained using ps pulses are more complex and stronger, including Al (marked by γ), Al_2_O_3_ (marked by χ), Al(OH)_3_ (marked by Ι), and Al_2_O_3_ 3H_2_O (marked by Δ). The main difference at fs ablation conditions is that the presence of Al(OH)_3_ was not detected in XRD image, probably due to weak intensity. Its composition was mainly a mixture of Al and Al_2_O_3_, and the content of Al_2_O_3_ was relatively larger.

Compared with the same aging stage of NPs obtained by different ablation conditions, it is easy to find that the NPs prepared by ps pulses exhibit larger size after aging and self-assembly, while the shorter wavelength laser sources play a positive role in obtaining smaller particle size distribution in the case of nanosecond pulses. Meanwhile, the NPs obtained by ultrashort pulses have better uniformity in morphology and narrower particle size distribution. In addition, the XRD data exhibiting the presence of Al NPs mainly for fs ablation could be interpreted in terms of direct nanoparticle yield via phase-explosion mechanism, while for ns and ps ablation, laser–plume interaction could destroy parent ablation products until atoms and ions with the following recondensation [62].

## 4. Theoretical Analysis of Nanoparticles Formation during Ablation of Bulk Aluminum in Water

### 4.1. Model

**Figure 7:**
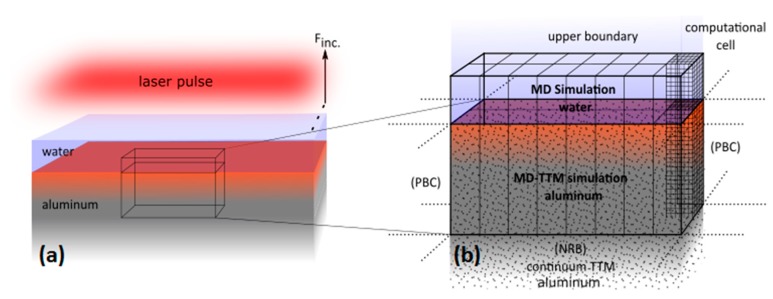
Computational box for simulations of laser formation of the Al NPs in water relays on the experimental geometry for the case of large laser spot size when the laser intensity across the computational cell can be considered uniform (**a**). The total computational cell is schematically represented for multiprocessing mode in (**b**). In each particular processor core, an atomistic-continuum molecular dynamics (MD)-two temperature model (TTM) for metal part and ordinary MD model for water part are solved in 3D space (internal mesh is shown for one processor core). Beneath the non-reflective boundaries (NRB), an ordinary TTM model is solved in continuum to account for the heat flux outward metal surface region describing the cooling process. By analogy, the NRB boundaries atop the water layer mimic the infinitely thick water layer, above which only mechanical properties of water are considered in continuum.

The idea of modeling of nanoparticles generation in water on the experimental scale is schematically presented in Figure 7a. The laser spot size on the metal surface is considered large (0.05–0.3 mm) as compared to the lateral size of the modeling cell (100 nm). Therefore, the laser intensity distribution in lateral X and Y directions can be considered uniform. For instance, dealing with the laser spot size of ~50 μm in diameter, its central part will not be significantly affected by the process of thermal diffusion in radial direction up to ~1 μs. This time is estimated from our preliminary calculations with the TTM approach [47]. Since this time can be considered as long enough for the processes of formation and solidification of the nanoparticles [51], it gives us a possibility to impose periodic boundary conditions (PBC) in X and Y directions for the whole simulation time up to 1 ns. Accounting for the finite size of the generated NPs (10–50 nm) in the experiments, for the direct comparison of the simulation results with the experimental data, the total computational cell for large scale parallel simulations is schematically shown in Figure 7b.

To avoid expensive and unnecessary MD integrations in the deep bulk of aluminum, the dynamically behaving non reflective boundary (NRB) conditions in Z direction were imposed at the depth of 400 nm. The NRB conditions are described in Reference [63] and the case of modeling of nanostructuring processes are modified in Reference [51]. The NRB conditions are designed to absorb the laser-generated pressure wave, which unloads the internal stresses in the vicinity of the surface due to laser heating. Dictated by the research interest, the MD-TTM model was applied only above that limit in the assumption that no phase transition would take place beneath the NRB. This assumption was verified in all our simulations. However, the NRB conditions are transparent for the heat flux, and the ordinary TTM model was accounting for the electron and phonon temperature dynamics on the scale up to 50 µm below the irradiated surface. Also, atop the aluminum target, a water layer of 500 nm thickness was placed, ending with NRB conditions (with parametrization for water) as well, thus representing an infinitely thick water layer. The size of 500 nm was chosen based on preliminary calculations and has to accommodate the laser-induced processes resulting in the ablation of Al, formation of the NPs, and their solidification process, taking place inside the total computational cell. Thus, the aluminum–water total computational cell consisting of ~300,000,000 atoms was taken with dimensions of 75 × 100 × 900 nm in X, Y, and Z directions, respectively, with an atomic resolution of the metal part within 400 nm below the surface and water part within 500 nm above.

For a possibility of the direct comparison of our simulation results with the experimental data, the atomistic-continuum MD-TTM model implements a realistic embedded atom method (EAM) interatomic potential for aluminum optimized by Zhakhovskii et al. [64]. This potential reproduces the experimental thermophysical properties of aluminum (such as equilibrium melting temperature, heat capacity, volume of melting, and linear thermal expansion coefficient) with an accuracy of more than 99.5% [65]. The modeling of water, however, is a more challenging task. Due to high polarity of the water molecule, the existing water models [66,67,68], although nicely representing some of its properties, suffer from one or another limitation, depending on the particular simulation task.

Based on general concepts of the possible laser-induced mechanisms involved into the formation of NPs during the laser ablation of aluminum under spatial confinement due to a water layer, in this work we used newly designed EAM water potential by Zhakhovskii [69], which primarily reproduces its mechanical properties with an accuracy of more than 99%. Although missing the description of any chemistry involved into the laser ablation process, the simplicity of such potential, however, helps us to overcome too shallow integration time-step, as it is in the explicit water model [66], and the system size limitations [67]. Also, unlike to the model presented in Reference [68], the chosen water model representation [64] allows for a direct interaction between water and metal particles as the interaction between single molecules (atoms).

Furthermore, in these simulations, we do not consider any optical effects such as refocusing of the laser beam and absorption processes in water. In other words, we consider the water layer as absolutely transparent, serving the purpose of the spatial confinement only. The relation between the incident and the absorbed fluencies, therefore, is based on the reflectivity function of aluminum and is taken from tabulated values of extinction coefficients [70] for the given wavelength of 1064 nm. This choice is justified by the fact that the pulse duration of 200 ps (same for 50 ps) is much longer than the characteristic electron–phonon relaxation time for aluminum (~1.5 ps) that excludes the development of a strong electron–phonon nonequilibrium and significant elevation of the electronic temperatures towards the Fermi values (~54,000 K), when the optical properties of the irradiated material can significantly deviate from their equilibrium values. Furthermore, to save the computational resources, we use the pulse duration of 50 ps in our simulations as it is still much longer than τe-ph and will not give any sensible difference from the point of NPs formation by 200 ps laser pulse. Finally, in our calculations, we do not consider any chemistry related to oxidation of Al NPs, since explicit consideration of the material electron density change and the corresponding modification of the interatomic potential would require the application of complex density functional theory (DFT) approaches of high computational cost. Although there are simplified descriptions of the chemical reactions within the classical MD approach [71], we leave this step for our future investigations.

Accounting for the assumptions above, the suggested computational approach is aimed at identification of general effects involved into the NPs generation process, solely caused by the mechanical and thermophysical action of a water layer. Such a general approach, however, is an important intermediate step toward the study of colloidal liquids evolution under different laser pulse regimes.

### 4.2. Results of Modeling and Discussion

**Figure 8:**
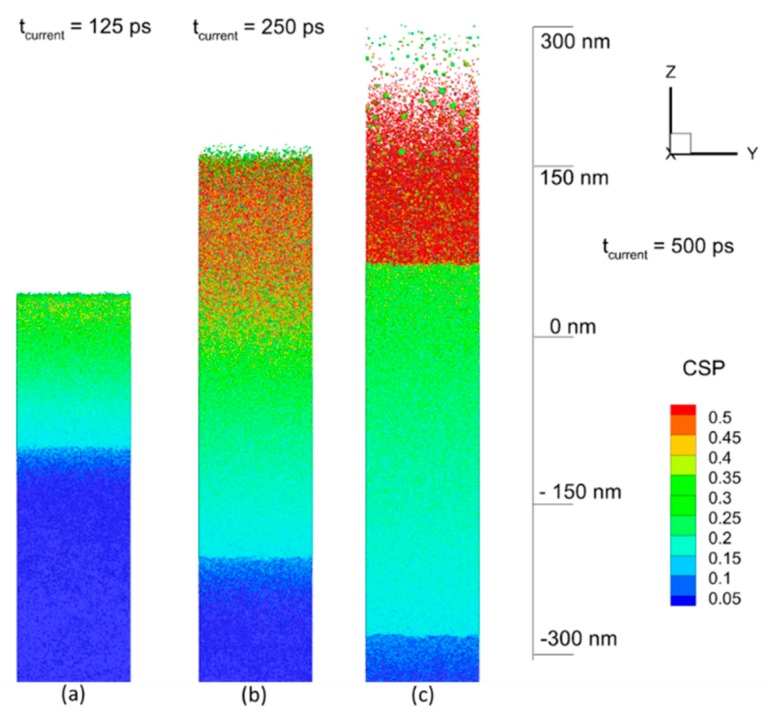
The result of 50 ps laser pulse interaction with Al in water at the working wavelength of 1064 nm and the incident fluence of 10 J/cm^2^ is shown as the sequence of atomic snapshots for 125 ps (**a**), 250 ps (**b**), and 500 ps (**c**). The atoms are colored by central symmetry parameter (CSP) for identification of local atomic structure: Solid < 0.08 < defects < 0.12 < liquid < 0.25 < surface < 0.50 < vapor. The water atoms are blanked in all snapshots for a better visualization of the ablation process.

The results of modeling of the NPs generation in water media due to a 50 ps laser pulse interacting with a thick Al target at the wavelength of 1064 nm and the incident fluence of 10 J/cm^2^ (the corresponding absorbed fluence was measured at the value of 0.385 J/cm^2^) can be seen in Figure 8a–c as a sequence of sliced atomic snapshots of the total computational cell in YZ plane, taken at the times of 125 ps, 250 ps, and 500 ps from the begging of the pulse. The pulse with the duration much longer than the characteristic electron–phonon relaxation time cannot induce a strong electron–phonon nonequilibrium. The electronic temperature, therefore, does not reach high values and limited roughly within 10,000 K, when the laser-deposited energy dissipation channel through the electron thermal conduction is much weaker than through the electron–phonon collisions (lattice heating) [72]. This results in the establishment of thermal confinement regime, and the incurred damage by the target, therefore, has a purely thermal character [73]. Meanwhile, the material ablated from the surface region also removes a large part of thermal energy, and the solid–liquid interface (the melting front) comes to the rest by the time of 500 ps at the depth of −280 nm with respect to the position of the initial surface, as shown in Figure 8c.

**Figure 9:**
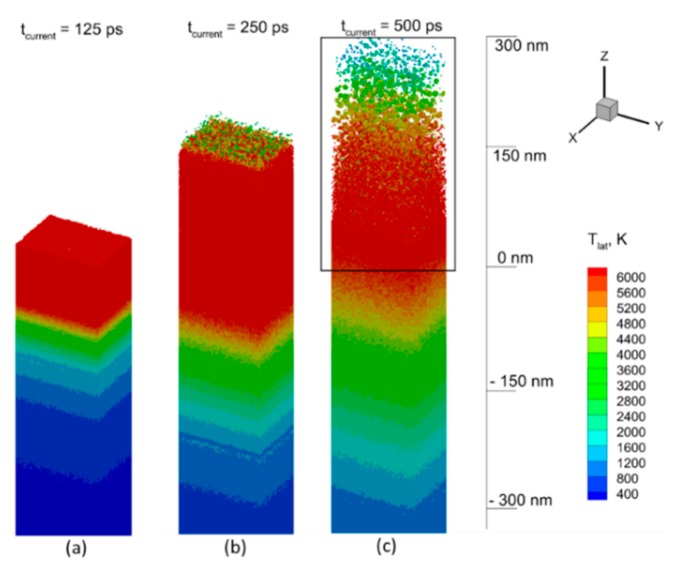
The lattice temperature field is shown for simulation times of 125 ps (**a**), 250 ps (**b**), and 500 ps (**c**). The water media is blanked here for visualization of metallic part of the computational cell. The rectangular region in (**c**) is zoomed for a more detailed observation in Figure 10.

For the thermophysical analysis of the induced process, one can consider the lattice temperature field, shown in Figure 9a–c, for the same times: 125 ps, 250 ps, and 500 ps. Each point in this Figure 9 represents a group of atoms (~2500 atoms) for which the local statistical distribution allows us to define temperature. Thus, one can see that during the laser energy deposition, the long heating time results in reaching the critical temperature value of more than 6000 K for the lattice, as shown in Figure 9a, and the ablation process is driven by the explosive boiling mechanism [74], the onset of which leads to the lift-off a number of molten material droplets, as shown in Figure 9b. These droplets, however, lose both the lift-off speed and the high temperature already at a distance of 300 nm from the initial surface, as shown in Figure 9c. The water media, having both the atomic density and the heat capacity comparable with those of metal, causes a significant resistance and efficient cooling to the moving upward ablated material and the ablation plume, therefore, is much smaller here as compared to that in vacuum [75].

**Figure 10:**
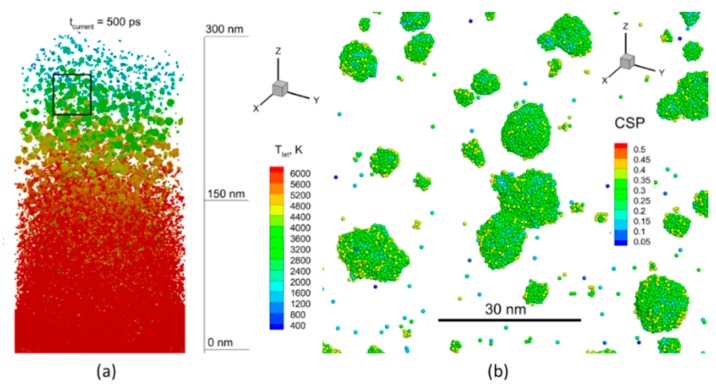
The zoomed region from Figure 9c allows for visualization of the top of ablation plume in the field of lattice temperature (**a**). The squared region is zoomed in (**b**) and represents the atomic view for examination of morphology of the forming Al NPs in water. The light blue atoms are indication of the onset of the solidification process. The water atoms are not shown.

The effect of spatial confinement by a water layer on the processes of nanostructuring of metals was investigated in [76] and can be reconfirmed here for the case of NPs generation in liquid media (water). First, the water spatial confinement leads to a significant suppression of the ablation plume or the growing nanofeature that makes the final surface smother as compare to that obtained in vacuum. Second, due to high heat capacity and its vaporization heat losses, the water media itself and its intensive evaporation process efficiently cool down the forming NPs that facilitates their rapid solidification after 500 ps, as shown in Figure 9c. This also can be seen in Figure 10a, where the lattice temperature filed is zoomed for the time of 500 ps and reveals a strong temperature drop in the direction of material ejection (ablation plume). Noticeably, the smaller NPs at the very top of the ablation plume are completely formed and have the temperature already below the melting point (933 K), while the deeper and bigger NPs reveal the onset of solidification process, zoomed in Figure 10b and shown at the atomic level. One can see that atomic structures on the nanoparticles’ surface have an ordered character, which indicates the beginning of crystallization process.

The effect of water media also results in a more uniform size distribution of the generated NPs, which is also very different from the results recently obtained in modeling of laser-induced NPs generation with short laser pulses in vacuum [76]. The formed NPs have more or less spherical shape, which, unlike the mechanical mechanism of ablation (spallation), originates from the relatively slow boiling mechanism, coalescing of the smaller droplets into the spherical particles (benefiting from the surface energy), and their fast re-solidification process at a certain distance from the initial surface. The subsequent elevation speed of the forming nanoparticles in water media is one order of magnitude slower than that in vacuum, so that by approximately ~1 ns of the simulation time, the motion of newly formed NPs is driven exclusively by the established hydrodynamic motion in water rather than by the initial momentum obtained due to the ablation process.

Finally, the already-formed NPs in the colloidal liquid can continue growing due to slow diffusion of Al atoms in water and can coalesce further into unusual structures, like those that can be seen in Figure 4. Apparently, the slow diffusion process facilitates the formation of NPs with highly pronounced effect of their crystal structure, as shown in Figure 10b. Similar to a salt crystal growing due to desorption process, the unusual form of nanoparticles and their connections are therefore owing to both the crystal structure of the corresponding elements and energetically favorable position of those element with respect to each other.

## 5. Conclusions

The ablation of Al bulk in distilled water using pulsed lasers with different pulse durations and wavelengths was systematically analyzed. The experimental results show that almost all of the ablation products obtained at different conditions contain Al, Al_2_O_3_, and Al(OH)_3_. It was found that the dynamics of NPs formation and modification can be divided into the formation, great change, and stable stages. The final shapes of NPs presented at the stabilization period after aging are analyzed using optical and morphology methods.

The initial NPs prepared by various pulse durations (5 ns, 200 ps, and 30 fs) showed regular spherical structures. Under the condition of 5 ns pulses with 1064 nm, morphology and particle size were similar during the aging process. The main components were the mixtures of Al, Al_2_O_3_, and Al(OH)_3_. Meanwhile, in the case of ns, 355 nm pulses, aggregation during aging significantly increased the sizes of NPs. Those NPs mainly consisted of Al(OH)_3_ with a small amount of Al and Al_2_O_3_. The NPs obtained by ablation using ps pulses exhibited conical structures dominated by Al_2_O_3_·3H_2_O and rod-like structures dominated by Al(OH)_3_. Compared with the former (ns) case, NPs with small size and uniform distribution were obtained during ablation using ps pulses. The main components of these NPs were Al and Al_2_O_3_. The application of fs pulses led to formation of granular NPs.

The process of NPs generation during laser ablation was theoretically studied using MD-based method on the example for 50 ps, 1064 nm pulses interacting with Al target in water media. The suggested combined atomistic-continuum MD-TTM model enabled the possibility of running the simulations on the experimental scale, which allowed direct comparison of the modeling results with the experimental data. It was identified that the main driving mechanism of the material ejection (ablation) originates from the explosive boiling due to laser heating of the material to the critical point and above. The material ejection occurs under conditions of thermal confinement so that the bigger part of the laser deposited energy remains in the vicinity of the target surface, therefore increasing the efficiency of the NPs formation. Along with parameters of the laser irradiation, the mechanical and thermophysical effects of the water media, to a large extent, have been found to play a determining role on the size distribution and morphology of the synthesized NPs. The additional resistance of the water media results in a suppression of the growing ablation plume, facilitating the uniform size distribution of NPs, whereas their heat losses due to large heat capacity of water and the thermal energy spent for water vaporization result in fast solidification of the produced NPs.

Our study has shown that pulsed laser ablation in a liquid environment has broad prospects, since it is feasible to selectively synthesize NPs, nanoparticle oxides, or hydroxides with specific morphology, size, and composition by adjusting external conditions, such as pulse width and wavelength of heating radiation. The knowledge of the dependence of the morphology and particle sizes on the pulse duration and other external conditions allows synthesizing nano-sized particles with specific morphology and properties for scientific and industry needs.

## Figures and Tables

**Table 1:** Summary of energy dispersive spectroscopy (EDS) measurements.

	Wt %		At %		Type	Picture
	Al	O	Al	O		
S_n (1064 nm)_	38.4	61.6	27	73	Al & Al_2_O_3_ & Al(OH)_3_	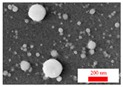
S_n (355 nm)_	35	65	24.2	75.8	Al & Al_2_O_3_ & Al(OH)_3_	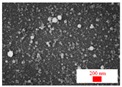
S_p_	32.4	67.6	22.1	77.9	Al_2_O_3_ 3H_2_O	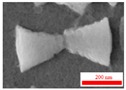
	18	82	11.5	88.5	Al(OH)_3_	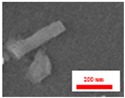
S_f_	36.3	63.7	25.8	74.2	Al & Al_2_O_3_	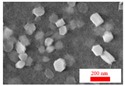

## References

[1] Pincella F., Isozaki K., Miki K. (2014). A visible light-driven plasmonic photocatalyst. Light Sci. Appl..

[2] Zeng H., Du X.W., Singh S.C., Kulinich S.A., Yang S., He J., Cai W. (2012). Nanomaterials via laser ablation/irradiation in liquid: A review. Adv. Func. Mater..

[3] Karabchevsky A., Mosayyebi A., Kavokin A.V. (2016). Tuning the chemiluminescence of a luminol flow using plasmonic nanoparticles. Light Sci. Appl..

[4] Kudryashov S.I., Samokhvalov A.A., Nastulyavichus A.A., Saraeva I.N., Mikhailovskii V.Y., Ionin A.A., Veiko V.P. (2019). Nanosecond-laser generation of nanoparticles in liquids: From ablation through bubble dynamics to nanoparticle yield. Materials.

[5] Gu M., Bao H., Gan X., Stokes N., Wu J. (2014). Tweezing and manipulating micro- and nanoparticles by optical nonlinear endoscopy. Light: Sci. Appl..

[6] Galfetti L., De Luca L.T., Severini F., Meda L.U., Marra G., Marchetti M., Regi M., Bellucci S. (2006). Nanoparticles for solid rocket propulsion. J. Phys. Condens. Matter.

[7] Tyagi H., Phelan P.E., Prasher R., Peck R., Lee T., Pacheco J.R., Arentzen P. (2008). Increased hot-plate ignition probability for nanoparticle-laden diesel fuel. Nano Lett..

[8] Chen X., Jia B., Zhang Y., Gu M. (2013). Exceeding the limit of plasmonic light trapping in textured screen-printed solar cells using Al nanoparticles and wrinkle-like graphene sheets. Light Sci. Appl..

[9] Zhou X., Fang Y., Zhang P. (2007). A new substrate for surface enhanced Raman scattering of dye Sudan molecules. Spectrosc. Acta Part A.

[10] Chowdhury M.H., Ray K., Gray S.K., Pond J., Lakowicz J.R. (2009). Al nanoparticles as substrates for metal-enhanced fluorescence in the ultraviolet for the label-free detection of biomolecules. Anal. Chem..

[11] Wang J.J., Walters F., Liu X., Sciortino P., Deng X. (2007). High-performance, large area, deep ultraviolet to infrared polarizers based on 40 nm line/78 nm space nanowire grids. Appl. Phys. Lett..

[12] Shimojo F., Ohmura S., Kalia R.K., Nakano A., Vashishta P. (2010). Molecular dynamics simulations of rapid hydrogen production from water using Al clusters as catalyzers. Phys. Rev. Lett..

[13] Czech E., Troczynski T. (2010). Hydrogen generation through massive corrosion of deformed Al in water. Int. J. Hydrogen Energy.

[14] Roach P.J., Woodward W.H., Castleman A.W., Reber A.C., Khanna S.N. (2009). Complementary active sites cause size-selective reactivity of Al cluster anions with water. Science.

[15] Zheng S., Fang F., Zhou G., Chen G., Ouyang L., Zhu M., Sun D. (2008). Hydrogen storage properties of space-confined NaAlH4 nanoparticles in ordered mesoporous silica. Chem. Mater..

[16] Balde C.P., Hereijgers B.P.C., Bitter J.H., de Jong K.P. (2008). Sodium alanate nanoparticles - linking size to hydrogen storage properties. J. Am. Chem. Soc..

[17] Yang Y., Wang S., Sun Z., Dlott D.D. (2004). Near-infrared laser ablation of poly tetrafluoroethylene (Teflon) sensitized by nanoenergetic materials. Appl. Phys. Lett..

[18] Henz B.J., Hawa T., Zachariah M.R. (2010). On the role of built-in electric fields on the ignition of oxide coated nano Al: Ion mobility versus Fickian diffusion. J. Appl. Phys..

[19] Yoon B., Häkkinen H., Landman U., Wörz A.S., Antonietti J.M., Abbet S., Judai K., Heiz U. (2005). Charging effects on bonding and catalyzed oxidation of CO on Au8 clusters on MgO. Science.

[20] Ganeev R.A., Singhal H., Naik P.A., Chakera J.A., Srivastava A.K., Dhami T.S., Joshi M.P., Gupta P.D. (2009). Influence of C60 morphology on high-order harmonic generation enhancement in fullerene-containing plasma. J. Appl. Phys..

[21] Yamamoto T. (2004). The relation between surface plasmon resonance and morphology of Ag nanodots prepared by pulsed laser deposition. Solid State Ion..

[22] Goh E.G., Xu X., McCormick P.G. (2014). Effect of particle size on the UV absorbance of zinc oxide nanoparticles. Scr. Mater..

[23] Fan G.-H., Qu S.-L., Guo Z.-Y., Wang Q., Li Z.-G. (2012). Size-dependent nonlinear absorption and refraction of Ag nanoparticles excited by fs lasers. Chin. Phys. B.

[24] Wang K., Long H., Fu M., Yang G., Lu P. (2010). Size-related third-order optical nonlinearities of Au nanoparticle arrays. Opt. Express.

[25] Lee S., Ahn A., Choi M.Y. (2012). Direct observation of aluminium ions produced via pulsed laser ablation in liquid: A ‘turn-on’ fluorescence study. Phys. Chem. Chem. Phys..

[26] Deng Z.-Y., Liu Y.-F., Tanaka Y., Ye J., Sakka Y. (2005). Modification of Al particle surfaces by c-Al2O3 and its effect on the corrosion behavior of Al. J. Am. Ceram. Soc..

[27] Deng Z.-Y., Liu Y.-F., Tanaka Y., Zhang H.-W., Ye J., Kagawa Y. (2005). Temperature effect on hydrogen generation by the reaction of c-Al2O3-modified Al powder with distilled water. J. Am. Ceram. Soc..

[28] Patil P.P., Phase D.M., Kulkarni S.A., Ghaisas S.V., Kulkarni S.K., Kanetkar S.M., Ogale S.B., Bhide V.G. (1987). Pulsed-laser-induced reactive quenching at liquid-solid interface: Aqueous oxidation of iron. Phys. Rev. Lett..

[29] Santillán J.M.J., Videla F.A., van Raap M.B.F., Schinca D.C., Scaffardi L.B. (2013). Analysis of the structure, configuration, and sizing of Cu and Cu oxide nanoparticles generated by fs laser ablation of solid target in liquids. J. Appl. Phys..

[30] Kim K.K., Kim D., Kim S.K., Park S.M., Song J.K. (2011). Formation of ZnO nanoparticles by laser ablation in neat water. Chem. Phys. Lett..

[31] Semaltianos N.G., Logothetidis S., Frangis N., Tsiaoussis I., Perrie W., Dearden G. (2010). Laser ablation in water: A route to synthesize nanoparticles of titanium monoxide. Chem. Phys. Lett..

[32] Acacia N., Barreca F., Barletta E., Spadaro D., Currò G., Neri F. (2010). Laser ablation synthesis of indium oxide nanoparticles in water. Appl. Surf. Sci..

[33] Musaev O.R., Yan J., Dusevich V., Wrobel J.M., Kruger M.B. (2014). Ni nanoparticles fabricated by laser ablation in water. Appl. Phys. A.

[34] Tilaki R.M., zad A.I., Mahdavi S.M. (2006). Stability, size and optical properties of silver nanoparticles prepared by laser ablation in different carrier media. Appl. Phys. A.

[35] Boardman A.D., Tsai D.P., Arboleda D.M., Santillán J.M.J., Herrera L.J.M., van Raap M.B.F. (2015). Structure, configuration, and sizing of Ni nanoparticles generated by ultrafast laser ablation in different media. Plasmon. Met. Nanostruct. Their Opt. Prop. XIII.

[36] Ganeev R.A., Baba M., Ryasnyansky A.I., Suzuki M., Kuroda H. (2004). Characterization of optical and nonlinear optical properties of silver nanoparticles prepared by laser ablation in various liquids. Opt. Commun..

[37] Jasbi N.E., Dorranian D. (2017). Dependence of laser ablation produced TiO_2_ NPs on the ablation environment temperature. Opt. Quantum Electron..

[38] Ishikawa Y., Shimizu Y., Sasaki T., Koshizaki N. (2006). Preparation of zinc oxide nanorods using pulsed laser ablation in water media at high temperature. J. Colloid Interface Sci..

[39] Solati E., Dorranian D. (2016). Effect of temperature on the characteristics of ZnO nanoparticles produced by laser ablation in water. Bull. Mater. Sci..

[40] Giorgetti E., Miranda M.M., Caporali S., Canton P., Marsili P., Vergari C. (2015). TiO_2_ nanoparticles obtained by laser ablation in water: Influence of pulse energy and duration on the crystalline phase. J. Alloys Compd..

[41] Dorranian D., Tajmir S., Khazanehfar F. (2013). Effect of laser fluence on the characteristics of Ag nanoparticles produced by laser ablation. Soft Nanosci. Lett..

[42] Shazia B., Shazaib K., Mahreen A., Nisar A., Umm K., Shahbaz A. (2015). Pulsed laser ablation of Ni in vacuum and N2 atmosphere at various fluencies. Quantum Electron..

[43] Nath A., Laha S.S., Khare A. (2011). Effect of focusing conditions on synthesis of titanium oxide nanoparticles via laser ablation in titanium–water interface. Appl. Surf. Sci..

[44] Tsuji T., Thang D.H., Okazaki Y., Nakanishi M., Tsuboi Y., Tsuji M. (2008). Preparation of silver nanoparticles by laser ablation in polyvinylpyrrolidone solutions. Appl. Surf. Sci..

[45] Sukhov I.A., Shafeev G.A., Voronov V.V., Sygletou M., Stratakis E., Fotakis C. (2014). Generation of nanoparticles of bronze and brass by laser ablation in liquid. Appl. Surf. Sci..

[46] Lasemi N., Pacher U., Zhigilei L.V., Bomatí-Miguel O., Lahoz R., Kautek W. (2018). Pulsed laser ablation and incubation of nickel, iron and tungsten in liquids and air. Appl. Surf. Sci..

[47] Anisimov S.I., Kapeliovich B.L., Perel’man T.L. (1974). Electron emission from metal surfaces exposed to ultrashort laser pulses. Zh. Eksp. Teor. Fiz.

[48] Ivanov D.S., Zhigilei L.V. (2003). Combined atomistic-continuum modeling of short-pulse laser melting and disintegration of metal films. Phys. Rev. B.

[49] Ivanov D.S., Lipp V.P., Blumenstein A., Kleinwort F., Veiko V.P., Yakovlev E., Roddatis V., Garcia M.E., Rethfeld B., Ihlemann J. (2015). Experimental and theoretical investigation of periodic nanostructuring of Au with UV laser near the ablation threshold. Phys. Rev. Appl..

[50] Ivanov D.S., Rethfeld B.C., O’Connor G.M., Glynn T.J., Volkov A.N., Zhigilei L.V. (2008). The mechanism of nanobump formation in femtosecond pulse laser nanostructuring of thin metal films. Appl. Phys. A.

[51] Ivanov D.S., Kuznetsov A.I., Lipp V.P., Rethfeld B., Chichkov B.N., Garcia M.E., Schulz W. (2013). Short laser pulse surface nanostructuring on thin metal films: Direct comparison of molecular dynamics modeling and experiment. Appl. Phys. A.

[52] Corkum P.B., Brunel F., Sherman N.K., Srinivasan-Rao T. (1988). Thermal response of metals to ultrashort-pulse laser excitation. Phys. Rev. Lett..

[53] Boltaev G.S., Ganeev R.A., Krishnendu P.S., Maurya S.K., Redkin P.V., Rao K.S., Zhang K., Guo C. (2018). Strong third-order optical nonlinearities of Ag nanoparticles synthesized by laser ablation of bulk silver in water and air. Appl. Phys. A.

[54] Rao K.S., Ganeev R.A., Zhang K., Fu Y., Boltaev G.S., Krishnendu P.S. (2018). Laser ablation–induced synthesis and nonlinear optical characterization of titanium and cobalt NPs. J. Nanopart. Res..

[55] Zhang K., Maurya S.K., Ganeev R.A., Rao K.S., Guo C. (2018). Ablated nickel NPs: Third harmonic generation and optical nonlinearities. J. Opt..

[56] Zeng H., Cai W., Li Y., Hu J., Liu P. (2005). Composition/structural evolution and optical properties of ZnO/Zn nanoparticles by laser ablation in liquid media. J. Phys. Chem. B.

[57] Barmina E.V., Kuzmin P.G., Shafeev G.A. (2011). Self-organization of hydrogen gas bubbles rising above laser-etched metallic aluminum in a weakly basic aqueous solution. Phys. Rev. E.

[58] Lee S., Jung H.J., Shin J.H., Choi M.Y. (2012). Production of size controlled Al and alumina nanoparticles via pulsed laser ablation in water. J. Nanosci. Nanotechnol..

[59] Lee S., Shin J.H., Choi M.Y. (2013). Watching the growth of Al hydroxide nanoparticles from Al nanoparticles synthesized by pulsed laser ablation in aqueous surfactant solution. J. Nanopart. Res..

[60] Stratakis E., Barberoglou M., Fotakis C., Viau G., Garcia C., Shafeev G.A. (2009). Generation of Al nanoparticles via ablation of bulk Al in liquids with short laser pulses. Opt. Express.

[61] Nikolov A.S., Atanasov P.A., Milev D.R., Stoyanchov T.R., Deleva A.D., Peshev Z.Y. (2009). Synthesis and characterization of TiOx nanoparticles prepared by pulsed-laser ablation of Ti target in water. Appl. Surf. Sci..

[62] Kudryashov S.I., Nastulyavichus A.A., Ivanova A.K., Smirnov N.A., Khmelnitskiy R.A., Rudenko A.A., Saraeva I.N., Tolordava E.R., Kharin A.Y., Zavestovskaya I.N. (2019). High-throughput laser generation of Si-nanoparticle based surface coatings for antibacterial applications. Appl. Surf. Sci..

[63] Schäfer C., Urbassek H.M., Zhigilei L.V., Garrison B.J. (2002). Pressure-transmitting boundary conditions for molecular dynamics simulations. Comp. Mater. Sci..

[64] Zhakhovskii V.V., Inogamov N.A., Petrov Y.V., Ashitkov S.I., Nishihara K. (2009). Molecular dynamics simulation of femtosecond ablation and spallation with different interatomic potentials. Appl. Surf. Sci..

[65] Gale W.F., Totemeier T.C. (2004). Smithell’s Metal. Reference Book.

[66] Nada H. (2003). An intermolecular potential model for the simulation of ice and water near the melting point: A six-site model of H2O. J. Chem. Phys..

[67] Dou Y., Zhigilei L.V., Postawa Z., Winograd N., Garrison B.J. (2001). Thickness effects of water overlayer on its explosive evaporation at heated metal surfaces. Nucl. Instrum. Methods B.

[68] Shih C.-Y., Wu C., Shugaev M.V., Zhigilei L.V. (2017). Atomistic modeling of nanoparticle generation in short pulse laser ablation of thin metal films in water. J. Colloid Interface Sci..

[69] Development of Interatomic EAM Potentials. https://www.researchgate.net/project/Development-of-interatomic-EAM-potentials.

[70] The World’s Sales Leader in Thin-Film Thickness Measurement. https://www.filmetrics.com.

[71] Kanski M., Garrison B.J., Postawa Z. (2016). Effect of oxygen chemistry in sputtering of polymers. J. Phys. Chem. Lett..

[72] Ivanov D.S., Rethfeld B.C. (2009). The effect of pulse duration on the character of laser heating: Photo-mechanical vs. photo-thermal damage of metal targets. Appl. Surf. Sci..

[73] Ivanov D.S., Lipp V.P., Veiko V.P., Jakovlev E., Rethfeld B., Garcia M.E. (2014). Molecular dynamics study of the short laser pulse ablation: Quality and efficiency in production. Appl. Phys. A.

[74] Zhigilei L.V., Lin Z., Ivanov D.S. (2009). Atomistic modeling of short pulse laser ablation of metals: Connections between melting, spallation, and phase explosion. J. Chem. Phys..

[75] Saraeva I.N., Kudryashov S.I., Rudenko A.A., Zhilnikova M.I., Ivanov D.S., Zayarnyi D.A., Simakin A.V., Ionin A.A., Garcia M. (2018). Effect of laser pulsewidth on fs/ps laser ablation of metals and silicon in air and liquids on nanoparticle yield and single-shot ablation thresholds. Appl. Surf. Sci..

[76] Ivanov D.S., Blumenstein A., Ihlemann J., Simon P., Garcia M.E., Rethfeld B. (2017). Molecular dynamics modeling of periodic nanostructuring of metals with a short UV laser pulse under spatial confinement by a water layer. Appl. Phys. A.

